# Cannabinoid 2 Receptor Activation Mitigates High-Fat Diet/Streptozotocin-Induced Nonalcoholic Fatty Liver Disease in Diabetic Mice by Modulation of Oxidative Stress, Inflammation, and Fibrosis

**DOI:** 10.1021/acsptsci.5c00610

**Published:** 2026-06-11

**Authors:** Hebaallah Mamdouh Hashiesh, M. F. Nagoor Meeran, Seenipandi Arunachalam, Sheikh Azimullah, Dhanya Saraswathiamma, Saeeda Al Marzooqi, Hanouf Al Shaka, Shamma Almehairbi, Abdulrahman Musabbeh Mohammed Alkaabi, Omar Shehab, Charu Sharma, Shreesh Ojha

**Affiliations:** † Department of Pharmacology and Therapeutics, College of Medicine and Health Sciences, 11239 United Arab Emirates University, Al Ain, P.O. Box-15551, UAE; ‡ Department of Medical Sciences, College of Medicine and Health Sciences, Khalifa University, Abu Dhabi, P.O. Box-15551, UAE; § Department of Pathology, College of Medicine and Health Sciences, 11239 United Arab Emirates University, Al Ain, P.O. Box: 15551, UAE; ∥ Department of Pediatrics, College of Medicine and Health Sciences, 11239 United Arab Emirates University, Al Ain, P.O. Box -15551, UAE; ⊥ Department of Genetics and Genomics, College of Medicine and Health Sciences, 11239 United Arab Emirates University, Al Ain, P.O. Box -15551, UAE; # Zayed Bin Sultan Center for Health Sciences, College of Medicine and Health Sciences, 11239 United Arab Emirates University, Al Ain, P.O. Box: 15551, UAE

**Keywords:** nonalcoholic fatty liver disease, β-caryophyllene, Nlrp3 inflammasome, Nrf2 activation, fibrosis

## Abstract

Nonalcoholic fatty liver disease (NAFLD) represents one of the multifactorial complications of type 2 diabetes (T2D). The cannabinoid type 2 receptor (CB2R) plays a role in diabetes and diabetic complications; therefore, the aim of the present study was to investigate the role of β-caryophyllene (BCP), a CB2R agonist, in NAFLD associated with T2D and underlying CB2R-mediated pharmacological and molecular mechanisms. The murine model of T2D was developed by feeding male C57BL/6 J mice a high-fat diet along with streptozotocin (STZ) injections. After developing diabetes, the animals were orally administered BCP (50 mg/kg, p.o.) for 12 weeks. Treatment with BCP reduced the elevation of liver injury markers and inhibited the expression of NADPH oxidase 2 and NADPH oxidase 4, activating Nrf2 signaling and showing liver protective effects and mitigation of oxidative stress. BCP treatment also inhibited hepatic inflammation, as shown by inhibition of NOD-like receptor protein 3 inflammasome activation in T2D mice. Furthermore, treatment with BCP suppressed hepatic fibrosis and endothelial-to-mesenchymal transition by inhibiting transforming growth factor-β/suppressor of mothers against decapentaplegic (Smad) signaling. Taken together, BCP appears to efficiently improve liver function in diabetic mice by suppressing pathologic events of oxidative stress, inflammation, and fibrosis. To reveal the CB2R-dependent mechanism of BCP, the diabetic animals were pretreated with a CB2R antagonist, AM630, which is expected to abrogate the protective effects of BCP. Pretreatment with AM630 abolished the beneficial effects of BCP on hepatic oxidative stress, inflammation, and fibrosis, as well as liver function in T2D mice. These results demonstrate that BCP has the potential to be a novel agent of natural origin from cannabinoids, like compounds, for NAFLD associated with diabetes. The study is suggestive of therapeutic benefits of BCP and could be useful in nutraceutical and pharmaceutical development of BCP and a new in-class agent mediating CB2R activation for NAFLD with diabetes.

## Graphical abstract

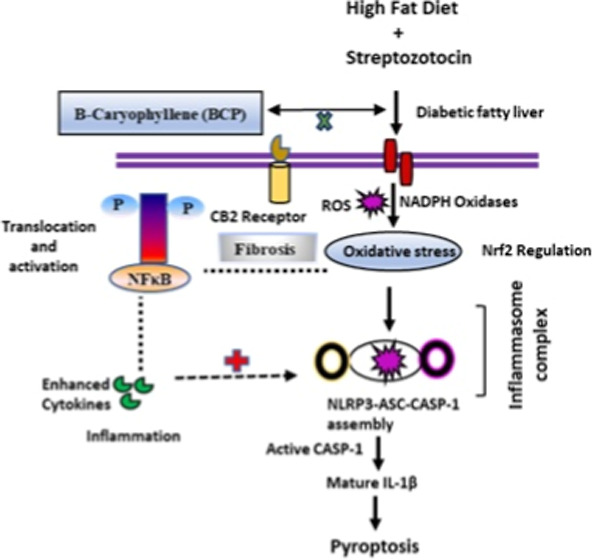

The occurrence of type 2 diabetes (T2D) mellitus accounts for over 95% of all diabetes patients, according to a published report.^1^ Despite the development of numerous therapeutic agents to manage diabetes, complications are on the rise, including liver diseases, particularly nonalcoholic fatty liver disease (NAFLD).^2^ Approximately one-fourth of the global adult population is affected by NAFLD, and about 70% of individuals with T2D also have NAFLD.^3^

The pathophysiology of T2D, concurrent with NAFLD, is complicated and not yet thoroughly recognized. A growing number of studies have demonstrated that enhanced oxidative stress plays an important role in initiating many complications linked with diabetes.^4,5^ According to earlier studies, there is a link between oxidative damage and NAFLD.^6^ In addition, uncontrolled oxidative stress enhances pro-inflammatory factor expression, thus encouraging inflammation.^7^ The overt generation of reactive oxygen species (ROS), the occurrence of high blood glucose levels with saturated fatty acids, facilitates the initiation and progression of numerous inflammatory cascades involving NOD-like receptor protein 3 (NLRP3) and its components.^8,9^ The role of NLRP3 in NAFLD progression has been demonstrated by the elevated expression of NLRP3, Caspase-1, and IL-1β in the liver tissue of nonalcoholic steatohepatitis mice.^10^ Furthermore, ROS and proinflammatory cytokines (TNF-α, IL-1β, and IL-6) are identified to stimulate hepatic stellate cells, which consequently cause sustained release of cytokines and overt generation of collagen. The hepatocytes and hepatic stellate cells, upon activation, produce one of the most potent profibrogenic cytokines, transforming growth factor-β (TGF-β), which mediates the expression of collagen I that plays a critical role in the development of hepatic fibrosis and severity of NAFLD.^11^ Considering the vital role of pathogenic events of oxidative damage, inflammation, and fibrosis in the liver, it is prudent that pharmacological targeting of these processes could be of therapeutic importance for the treatment of NAFLD in diabetes.^12^

In recent years, various pharmacological mechanisms and targets that play a vital role in hepatic oxidative damage, inflammation, and fibrosis have been explored to find novel therapeutic agents for NAFLD. Among various pharmacological targets, the endocannabinoid system—particularly cannabinoid receptor type 2 (CB2), which is predominantly expressed in immune cells such as macrophages—has emerged as an important focus in drug discovery, consistent with the fact that a large number of currently available drugs target G protein-coupled receptors.^13^ The CB2 receptors, G protein-coupled receptors present in liver fibrogenic cells and Kupffer cells, have been shown to play a significant role in liver injury and are believed to contribute to protective effects.^14,15^ The beneficial effects of CB2 receptor activation are primarily dependent on its mitigatory role on oxidative stress, inflammation, and fibrosis mediated by CB2 receptors located in hepatic immune cells and/or hepatic myofibroblasts, with paracrine effects on hepatocytes, which have no expression of CB2 receptors. In particular, CB2 receptor activation restricts the progression of liver fibrosis.^14^ A recent study in a murine model demonstrated that CB2 receptors, upon activation, diminish the proliferation of hepatic macrophages and prevent the release of inflammatory mediators in acute liver failure by downregulating HIF-1α, thereby inhibiting glycolysis.^16^ Importantly, CB2R deletion contributed to the progression of NAFLD via modulating the gut microbiome.^17^ These protective effects contrast with the profibrogenic role of CB1 receptors, suggesting that selectively targeting CB2R offers antioxidant, anti-inflammatory, and antifibrotic effects that translate into hepatoprotective benefits.^18^

Among numerous ligand types of endogenous, synthetic, and natural origin targeting CB2R, the ligands of natural origin generated special interest due to availability, accessibility, selectivity, and lack of psychotropic effects. β-Caryophyllene (BCP), chemically a natural bicyclic sesquiterpene predominantly found in large quantities in many essential oils of dietary plants from black pepper, cloves, and basil, is one of the agents of natural origin selectively targeting CB2R, termed a dietary cannabinoid. BCP was first identified as a functional CB2 receptor agonist with selective affinity for CB2R (*K*_i_ ≈ 155 ± 4 nM) and devoid of meaningful interaction with CB1 receptors, which renders it devoid of psychoactive effects.^19^ The functional assays demonstrated that BCP activates CB2R-mediated Gi and Go protein signaling, reduces intracellular cyclic AMP (cAMP), and exerts downstream anti-inflammatory and analgesic actions.^19^ BCP has received its safe status, classified as “generally regarded as safe” by the FDA in the food additive category for use as a flavor enhancer due to its favorable taste and aroma. The available data from numerous studies demonstrated that it has a wide safety margin and has negligible toxicity upon oral use. BCP is also found in marijuana/hemp (Cannabis spp.), the traditional source of cannabinoids and copaiba (Copaifera spp.), which have been used in ancient medicine, and their therapeutic benefits are attributed to anti-inflammatory properties.^20^ Beyond receptor binding, BCP has been validated in multiple preclinical disease models, where its protective effects were strictly dependent on CB2R activation. The wide availability and accessibility of dietary plants, negligible toxicity, multiple pharmacological properties, and their selective and specific targeting of CB2R, which makes them devoid of psychotropic effects, highlight their therapeutic potential for the treatment of numerous inflammatory diseases.^19^ BCP has been widely studied in numerous disease models, wherein it has been shown to exert CB2 receptor-mediated protective effects, including attenuation of pain,^21,22^ neuroinflammation,^23,24^ colitis,^25^ arthritis,^26^ and cardiovascular injury,^27,28^ as well as renal and hepatic injury.^29,30^ It was also shown to promote bone remodeling,^31^ wound healing,^32^ and treatment of asthma,^33^ and glioblastoma.^34^ The therapeutic benefits in these studies are largely driven by antioxidant, anti-inflammatory, and antiapoptotic mechanisms. Many of these studies demonstrated CB2R-mediated pharmacological mechanisms by pharmacological challenge with AM630, a CB2R antagonist that selectively abolishes the positive effects of CB2R agonist, BCP. The CB2R-mediated effects of BCP were also demonstrated in CB2 knockout mice, confirming receptor specificity. The role of BCP has been demonstrated in experimental models of diabetes and some of its complications. However, there are no data available on the role of BCP in NAFLD, one of the complex complications of T2D involving oxidative stress, inflammation, and fibrosis. Therefore, the current study was undertaken to examine the role of BCP on NAFLD in the murine model of high-fat diet (HFD)/streptozotocin-induced T2D, focusing on oxidative stress, inflammation, and fibrosis. A CB2R blocker, AM630, was used to demonstrate the CB2R-mediated pharmacological mechanisms.

Despite promising preclinical effects of selective CB2R agonists in many experimental models, no such agonists are available for clinical testing in liver diseases to date. Contrasting with the potent synthetic CB2R agonists, which have shown potential in animal models, the phytochemical BCP might be more immediately tested in humans, as it is an FDA-approved food additive, so it has instant translational potential.

## Materials and Methods

### Animals

The present study was approved by the Animal Ethics Committee of the United Arab Emirates University, United Arab Emirates (Approval Number: A-30-12). The mice were acquired from the animal research facility, College of Medicine at the United Arab Emirates University, UAE. Male C57BL/6 mice (eight weeks old, body weight 20–25 g) were used in the present study. The animals were housed in standard laboratory animal house conditions (22–25 °C, 60%–70% humidity, 12 h light/12 h dark cycle).

### Diabetes Induction and Experimental Design

T2D was induced according to our earlier study, following 1 week of acclimatization.^28,35^ After feeding a HFD (45% kcal from fat, no. D12451; Research Diets, USA) to mice for 4 weeks, a single injection of STZ (100 mg/kg/i.p.) dissolved in 0.1 M citrate buffer (pH 4.5) was administered. A similar amount of citrate buffer was injected into the normal control mice. The dose of BCP was selected based on previous studies.^28,36^ Meanwhile, normal control and BCP control mice received a vehicle and a standard chow diet, while HFD mice were fed only HFD throughout the experiment. Mice with fasting blood glucose levels of more than 250 mg/dl were regarded as diabetic and continued a HFD (45%) for another 12 weeks.

A total of 90 animals were randomly allocated into six experimental groups, each consisting of 15 mice. The groups were assigned from groups I to VI.

Group I: The animals in the normal control group received the vehicle (light olive oil) orally for 12 weeks.

Group II: The BCP group received BCP (50 mg/kg, orally) for 12 weeks.

Group III: The HFD group received the vehicle (light olive oil) orally for 12 weeks.

Group IV: T2D animals received a vehicle (light olive oil) orally for 12 weeks.

Group V: T2D animals received BCP (50 mg/kg orally) for 12 weeks.

Group VI: T2D animals were administrated AM630 (1.5 mg/kg/d, orally), a selective CB2R antagonist, 30 min before the oral treatment of BCP (50 mg/kg/d, orally) for 12 weeks.

## Blood Collection and Tissue Preparation

The animals were sacrificed after an overnight fast at the end of the 12 weeks of treatment protocol. The serum levels of lactate dehydrogenase (LDH), alanine transferase (ALT), and aspartate transaminase (AST) were detected with an automatic analyzer (Idexx Laboratories, Hoofddorp, The Netherlands). For histological analysis, the liver specimens were thoroughly dissected and preserved in 4% paraformaldehyde, whereas for Western blot and biochemical analysis, the samples were kept at −80 °C.

### Assessment of Oxidative Stress and Antioxidants

Malondialdehyde (MDA), a lipid peroxidation marker, was assessed in the liver tissue of mice utilizing a commercial kit (Northwest Life Sciences, USA). The contents of reduced glutathione (GSH) were evaluated by using a commercial GSH assay kit (Sigma-Aldrich, USA). In liver tissues, the estimation of activities of superoxide dismutase (SOD) and catalase was carried out using a commercial kit (Cayman Chemicals Co., USA).

### Analysis of Inflammatory Cytokines

The levels of IL-6, IL-1β, TNF-α, and IL-10 in liver tissues were measured using the following protocol supplied with ELISA kits procured from R&D Systems, USA. The results were expressed as pg/mL.

### Staining of Collagen Fibers in the Liver

To determine the collagen fiber, the liver sections underwent staining with Masson’s trichrome (ab150686; Abcam, Cambridge, UK) following the instructions provided by the manufacturer of the kit. Other sets of liver sections were stained with Picrosirius red staining (ab245887; Abcam, Cambridge, UK). The light microscopic changes were examined following hematoxylin and eosin staining and observations under a light microscope (BX41, Olympus). ImageJ software was used to quantify the results.

### Protein Extraction and Western Blotting

The liver tissue lysates were prepared in the RIPA buffer, and the protein concentration was measured by using the protein assay kit (Pierce BCA) procured from Thermo Fisher Scientific, USA. The samples were separated using SDS-PAGE gel electrophoresis and then shifted to a membrane composed of polyvinylidene difluoride. Thereafter, the membrane was blocked with 5% dry milk, nonfat in nature (1 h), and incubated with the designated primary antibody at 4 °C for a night (Table 1). Immunoreactive bands were identified using an HRP-linked secondary antibody, and quantitative protein band density was visualized and analyzed using ImageJ software. GAPDH and Lamin-B were used as loading controls for the estimation of total protein expression.

**Table 1:** Dilution, Catalogue, Manufacturer, and Source of Primary Antibodies Used

primary antibody	dilution	cat no	manufacturer	sources of species
TGF-β	1:1000	3711	Cell Signaling Technology	rabbit
a-SMA	1:500	14968	Cell Signaling Technology	rabbit
vimentin	1:1000	5741	Cell Signaling Technology	rabbit
P-Smad2/3	1:500	8828	Cell Signaling Technology	rabbit
T-Smad2/3	1:500	3102	Cell Signaling Technology	rabbit
collagen I	1:500	ab270993	Abcam	rabbit
collagen III	1:500	ab7778	Abcam	rabbit
fibronectin	1:500	ab2413	Abcam	rabbit
NOX4	1:1000	PA5-85479	Thermo Fisher Scientific	rabbit
Nrf2	1:1000	ab31163	Abcam	rabbit
Keap1	1:500	8047	Cell Signaling Technology	rabbit
HO-1	1:500	ab13243	Abcam	rabbit
SOD2	1:1000	13141	Cell Signaling Technology	rabbit
SOD3	1:1000	PA5-93329	Thermo Fisher Scientific	rabbit
GPX1	1:1000	PA5-26323	Thermo Fisher Scientific	rabbit
catalase	1:2000	14097	Cell Signaling Technology	rabbit
NLRP3	1:500	ab214185	Abcam	rabbit
ASC	1:500	sc-514414	Santa Cruz Biotechnology	mouse
IL-18	1:500	ab191860	Abcam	rabbit
GAPDH	1:2000	97166	Cell Signaling Technology	mouse
lamin-B	1:2000	12586	Cell Signaling Technology	rabbit

### Statistical Analysis

GraphPad Prism 8.0 software was used for data analysis, and the data obtained were expressed as mean ± standard error. In order to establish statistical differences between the groups, Tukey’s multiple comparison tests was employed in the data analysis. The criterion of statistical significance was set at *P* < 0.05.

## Results

### CB2R Agonist BCP Relieves Hepatic Damage in HFD/STZ-Induced Diabetic Mice

ALT, AST, and LDH are prevalent biomarkers of liver injury, and the data collected confirmed that the levels of ALT, AST, and LDH in serum were elevated in HFD mice and further markedly increased in T2D mice compared to the NC mice, suggesting lesions in hepatic tissue. BCP administration restored the changes in the liver damage markers. Nonetheless, AM630 treatment before BCP blocked the ameliorative effect of BCP on liver injury markers (Figure 1A–C). The above data showed that HFD/STZ could result in hepatic damage in T2D mice, and BCP administration could ameliorate this condition via CB2R activation.

**Figure 1:**
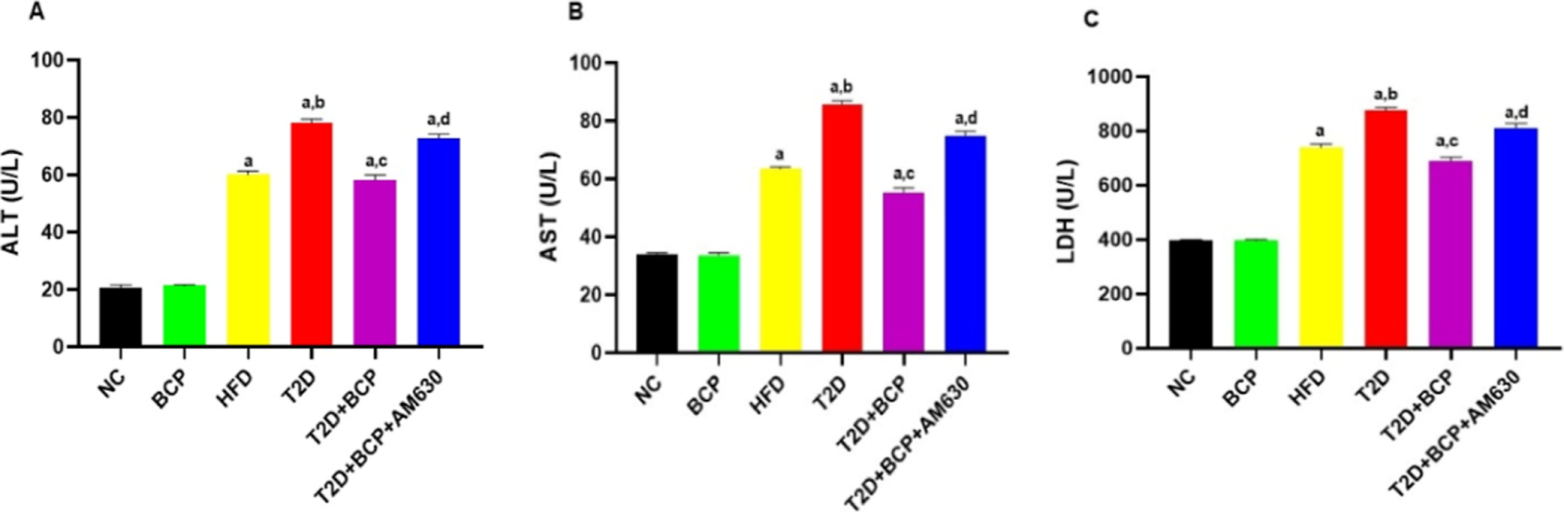
Effect of BCP treatment on liver injury markers in HFD/STZ-induced mice. (A) ALT. (B) AST. (C) LDH. Data are expressed as mean ± SEM (*N* = 8). Statistical analysis was performed using one-way analysis of variance (ANOVA), followed by Tukey’s post hoc test. ^a^ *P* < 0.05 compared to NC, ^b^ *P* < 0.05 compared to HFD, ^c^ *P* < 0.05 compared to T2D, ^d^ *P* < 0.05 compared to T2D + BCP.

### CB2R Agonist BCP Ameliorates Hepatic Oxidative Stress in HFD/STZ-Induced Diabetic Mice

We observed that following HFD feeding, the concentration of GSH, as well as the activities of SOD and catalase in the hepatic tissue of mice, all reduced remarkably. At the same time, MDA was increased compared with the NC group. The T2D mice further displayed augmented hepatic oxidative stress, which was mitigated by BCP treatment. AM630, a CB2R blocker administered before BCP treatment, has significantly abolished the positive activities of BCP on oxidative stress and antioxidants (Figure 2A–D).

**Figure 2:**
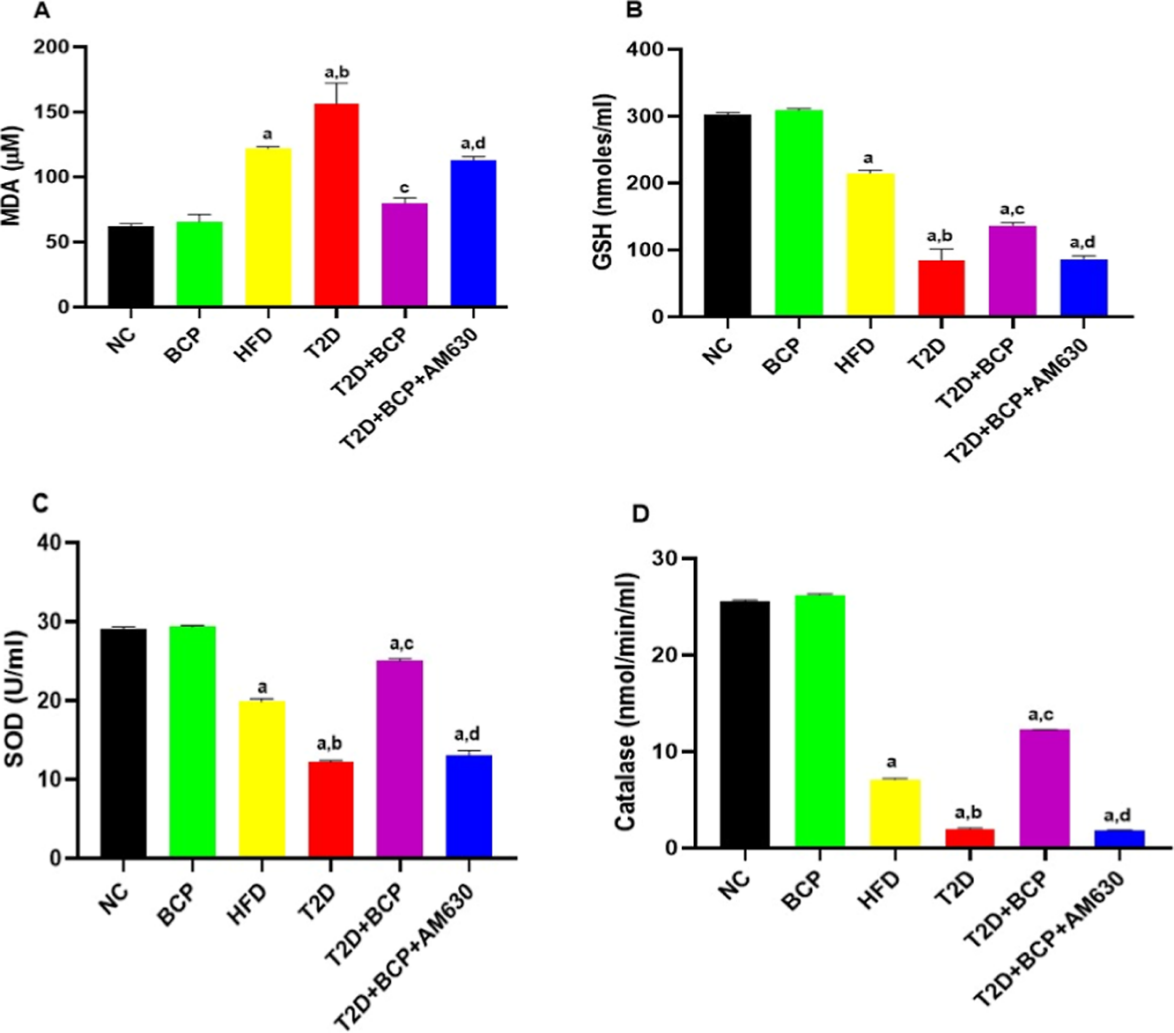
Effect of BCP treatment on oxidative stress and antioxidant enzymes in HFD/STZ-induced mice. (A) MDA. (B) GSH. (C) SOD. (D) Catalase. Data are expressed as mean ± SEM (*N* = 8). Statistical analysis was performed using one-way ANOVA, followed by Tukey’s post hoc test. ^a^ *P* < 0.05 compared to NC, ^b^ *P* < 0.05 compared to HFD, ^c^ *P* < 0.05 compared to T2D, ^d^ *P* < 0.05 compared to T2D + BCP. MDA, Malondialdehyde; GSH, reduced glutathione; SOD, superoxide dismutase.

### CB2R Agonist BCP Ameliorates Hepatic Oxidative Damage in HFD/STZ-Induced Diabetic Mice via the Nrf2 Signaling Pathway

The Nrf2 signaling pathway has a critical role in protecting hepatic tissues from oxidative stress. When compared with the NC mice, Western blot data revealed reduced expression of Nrf2, heme oxygenase-1 (HO-1), NAD­(P)H quinone dehydrogenase 1 (NQO1), GSH peroxidase 1 (GPX1), SOD1, SOD2, and catalase in HFD mice and further decreased in T2D mice with additional decrease in SOD3 expression, whereas the expression of Keap1 was improved in the liver tissues of the animals received HFD and further increased in T2D mice. Also, T2D mice demonstrated enhanced hepatic oxidative damage, which was shown by an enhanced expression of NOX2 and NOX4 compared with NC mice. BCP treatment produced a remarkable rise in the hepatic protein expression of Nrf2, HO-1, NQO1, GPX1, SOD1, SOD2, SOD3, and catalase; it also caused a remarkable fall in the expression of Keap1, NOX2, and NOX4 proteins in the liver tissues of T2D mice (Figure 3A,B). Importantly, the nuclear level of Nrf2 decreased in the T2D mice but significantly improved by BCP treatment. In contrast, the increased cytosolic Nrf2 level was restored after BCP treatment, thus proposing that BCP stimulated the translocation of Nrf2 from cytoplasm to nuclei (Figure 3 C,D). However, administration of AM630 before BCP treatment in T2D mice resulted in a noticeable reduction in BCP’s protective effects. Taken together, BCP activated Nrf2 signaling in a CB2R-dependent manner. This led to a decrease in hepatic oxidative stress in T2D animals.

**Figure 3:**
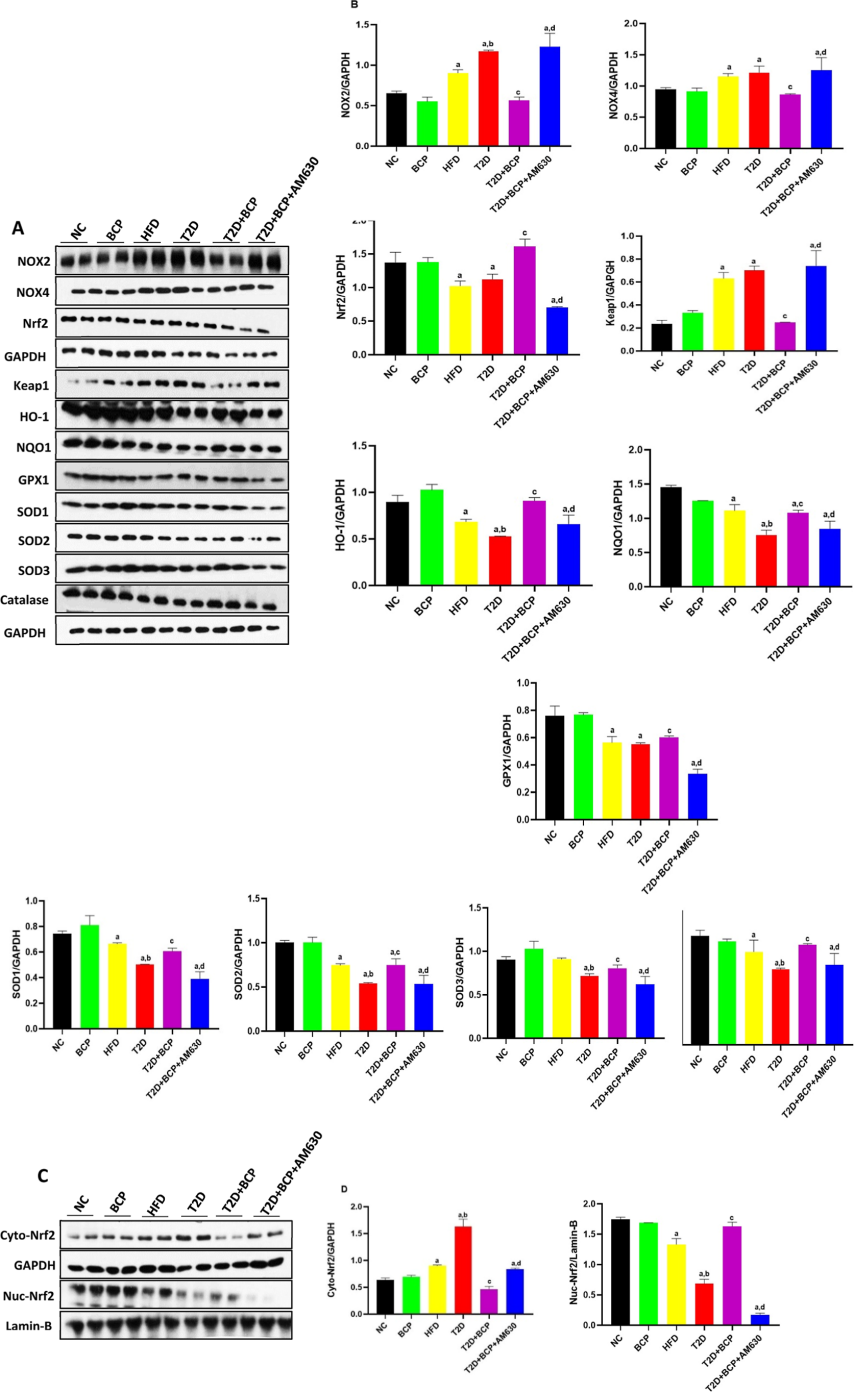
Effect of BCP treatment on hepatic oxidative damage and Nrf2 signaling in HFD/STZ-induced mice. (A) Western blotting analysis of NOX2, NOX4, Nrf2, Keap1, HO-1, NQO1, GPX1, SOD1, SOD2, SOD3, and catalase. (B) Densitometric analysis of hepatic protein expressions of NOX2, NOX4, Nrf2, Keap1, HO-1, NQO1, GPX1, SOD1, SOD2, SOD3, and catalase. (C) Western blotting analysis of cytoplasmic and nuclear Nrf2. (D) Densitometric analysis of hepatic protein expressions of cytoplasmic and nuclear Nrf2. Data are expressed as mean ± SEM (*N* = 3). Statistical analysis was performed using one-way ANOVA, followed by Tukey’s post hoc test. ^a^ *P* < 0.05 compared to NC, ^b^ *P* < 0.05 compared to HFD, ^c^ *P* < 0.05 compared to T2D, ^d^ *P* < 0.05 compared to T2D + BCP. NOX2, NADPH oxidase 2; NOX4, NADPH oxidase 4; Nrf2, nuclear factor erythroid 2-related factor 2; Keap1, kelch-like ECH-associated protein 1; HO-1, heme oxygenase-1; NQO1; NAD­(P)H quinone dehydrogenase 1; GPX1; glutathione peroxidase 1; SOD, superoxide dismutase.

### CB2R Agonist BCP Inhibits Hepatic Inflammation in HFD/STZ-Induced Diabetic Mice

To evaluate the role of BCP on inflammation in T2D mice, the levels of anti-inflammatory mediator, IL-10, as well as inflammatory mediators such as TNF-α, IL-6, and IL-1β were assessed in hepatic tissues. As depicted in Figure 4A–D, the concentration of TNF-α, IL-6, and IL-1β in the liver tissues was noticeably increased in HFD mice compared to NC mice and further elevated in T2D mice, while the hepatic level of IL-10 was decreased in HFD mice and further reduced in T2D mice compared to the NC group. Treating T2D mice with BCP significantly diminished the release of proinflammatory cytokines, along with a remarkable increase in the level of anti-inflammatory cytokine, IL-10, an effect that was nullified by prior treatment with AM630.

**Figure 4:**
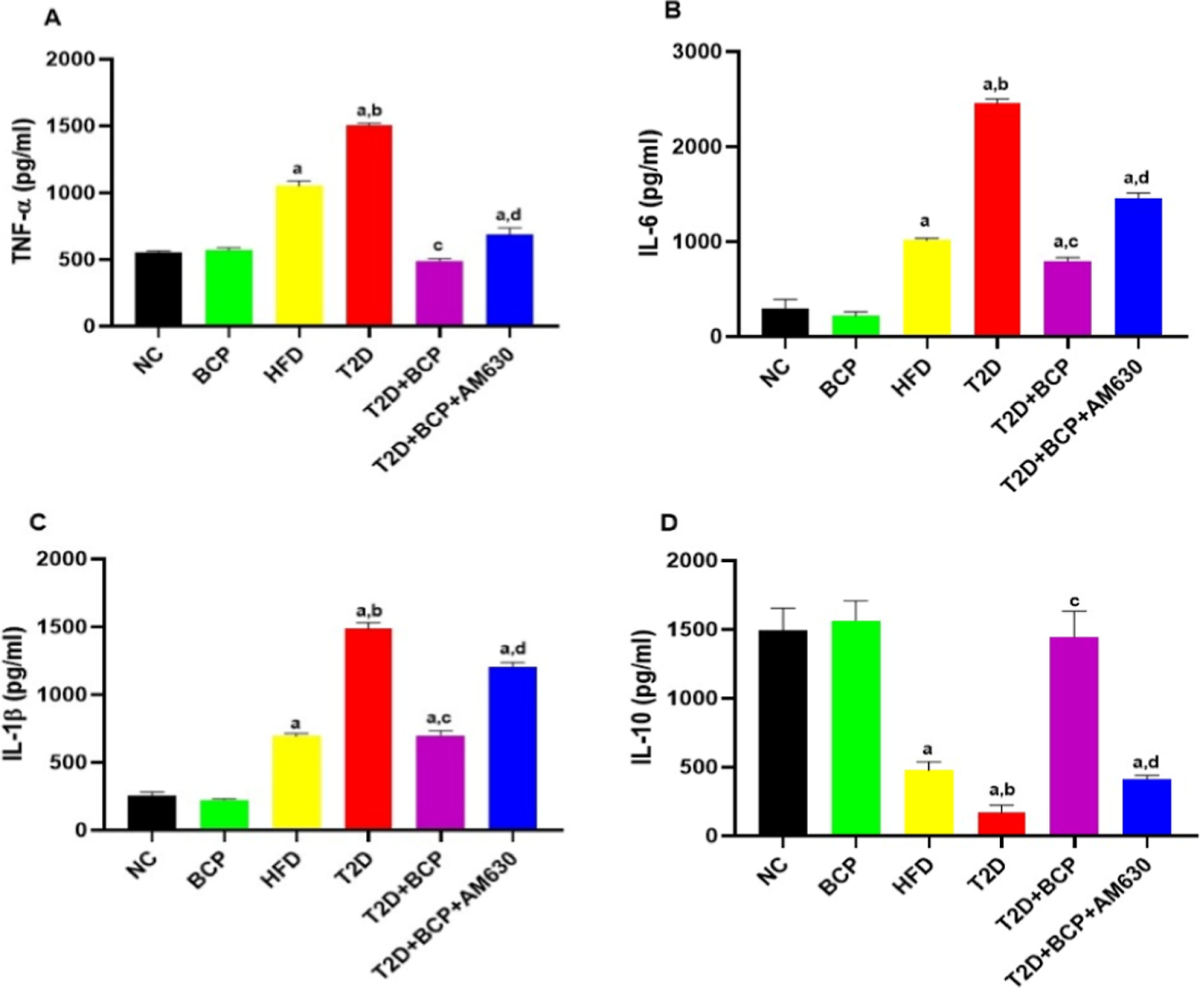
Effect of BCP on proinflammatory and anti-inflammatory markers in HFD/STZ-induced mice (A) TNF-α, (B) IL-6, (C) IL-1β, and (D) IL-10. Data are expressed as mean ± SEM (*N* = 8). Statistical analysis was performed using one-way ANOVA, followed by Tukey’s post hoc test. ^a^ *P* < 0.05 compared to NC, ^b^ *P* < 0.05 compared to HFD, ^c^ *P* < 0.05 compared to T2D, ^d^ *P* < 0.05 compared to T2D + BCP. TNF-α, Tumor necrosis factor-α; IL-6, interleukin-6; IL-1β, interleukin-1β; IL-10, interleukin-10.

### CB2R Agonist BCP Reduces NLRP3 Inflammasome Activation in HFD/STZ-Induced Diabetic Mice

It has become increasingly obvious that NLPR3 inflammasome activation is a contributing factor to the development of NAFLD. Western blot analyses revealed that the expression of NLRP3, ASC, and IL-18 was markedly improved in the hepatic tissues of HFD mice as well as in T2D mice. However, treatment with BCP resulted in a significant downregulation of NLRP3, ASC, and IL-18 expressions in T2D mice, as depicted in Figure 5A,B. Treatment with AM630 before BCP in T2D mice negated the positive effect of BCP on NLRP3 inflammasome activation. Taken together, BCP-induced CB2R activation has the potential to counteract NLRP3 inflammasome activation in T2D mice.

**Figure 5:**
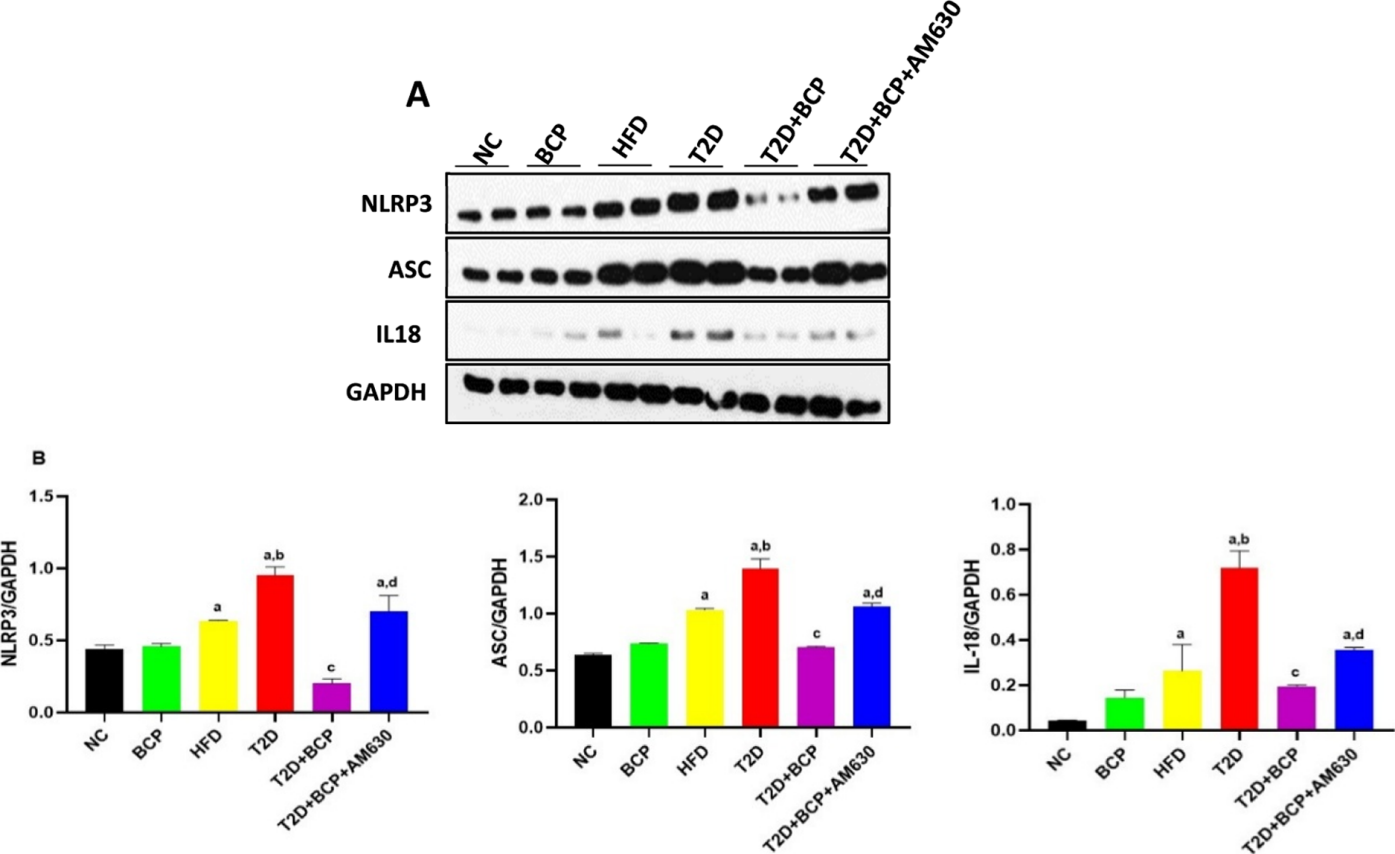
Effect of BCP treatment on hepatic NLRP3 inflammasome in HFD/STZ-induced mice. (A) Western blotting analysis of NLRP3, ASC, and IL-18. (B) Densitometric analysis of hepatic protein expressions of NLRP3, ASC, and IL-18. Data are expressed as mean ± SEM (*N* = 3). Statistical analysis was performed using one-way ANOVA, followed by Tukey’s post hoc test. ^a^ *P* < 0.05 compared to NC, ^b^ *P* < 0.05 compared to HFD, ^c^ *P* < 0.05 compared to T2D, ^d^ *P* < 0.05 compared to T2D + BCP. NLRP3, Nucleotide-binding domain, leucine-rich-containing family, pyrin domain-containing-3; ASC, apoptosis-associated speck-like protein containing a CARD; IL-18, interleukin-18.

### CB2R Agonist BCP Suppressed Hepatic Fibrosis in HFD/STZ-Induced Diabetic Mice

The staining of the liver tissues following Picrosirius red and Masson’s trichrome in the HFD mice exhibited enhanced collagen accumulation, while the liver tissues of the T2D mice displayed a further elevation in collagen accumulation. However, the BCP administration efficiently diminished the deposited collagen in the hepatic tissues of the T2D mice (Figure 6A–D). The administration of AM630 in T2D mice before the BCP counteracted the hepatoprotective role of BCP evidence by reduced accumulation of collagen.

**Figure 6:**
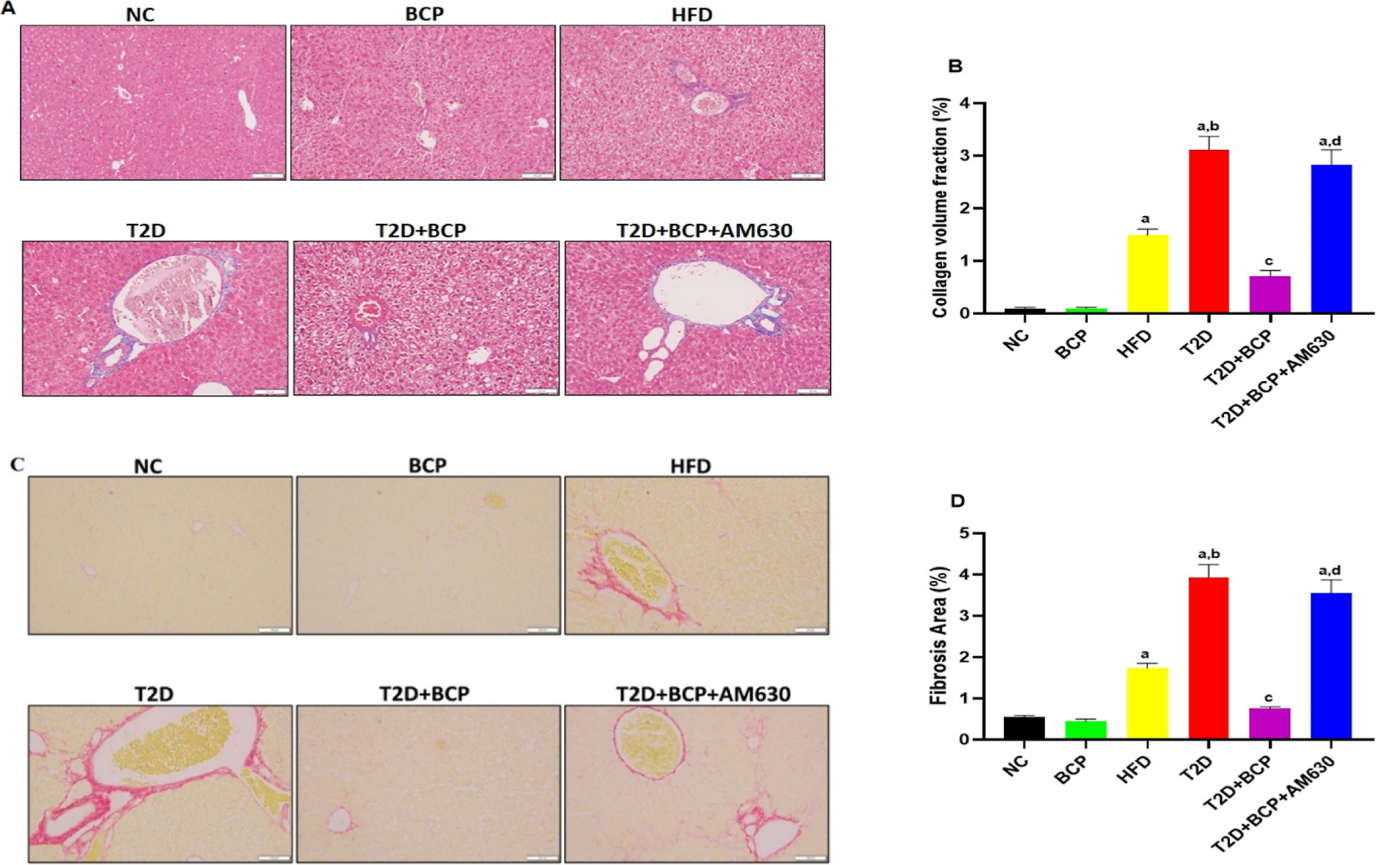
Effect of BCP treatment on hepatic fibrosis in HFD/STZ-induced mice. Hepatic fibrosis in HFD/STZ-induced mice is shown. (A) Masson trichrome staining (collagen is blue; 40× magnification). (B) Quantitative analysis of Masson trichrome staining (collagen volume fraction). (C) Picrosirius red staining (collagen fibers-stained bright red; 40× magnification). (D) Quantitative analysis of Picrosirius red staining (% fibrosis area). Data are expressed as mean ± SEM (*N* = 3). Statistical analysis was performed using one-way ANOVA, followed by Tukey’s post hoc test. ^a^ *P* < 0.05 compared to NC, ^b^ *P* < 0.05 compared to HFD, ^c^ *P* < 0.05 compared to T2D, ^d^ *P* < 0.05 compared to T2D + BCP.

The results of the Western blot displayed upregulated expressions of fibrotic markers (collagen I, collagen III, and fibronectin) in HFD mice (Figure 7A,B). The effects were more noticeable in the T2D mice, which were downregulated following treatment with BCP. In addition, we observed that the positive effects of BCP on the fibrotic markers were abrogated by pretreatment with AM630.

**Figure 7:**
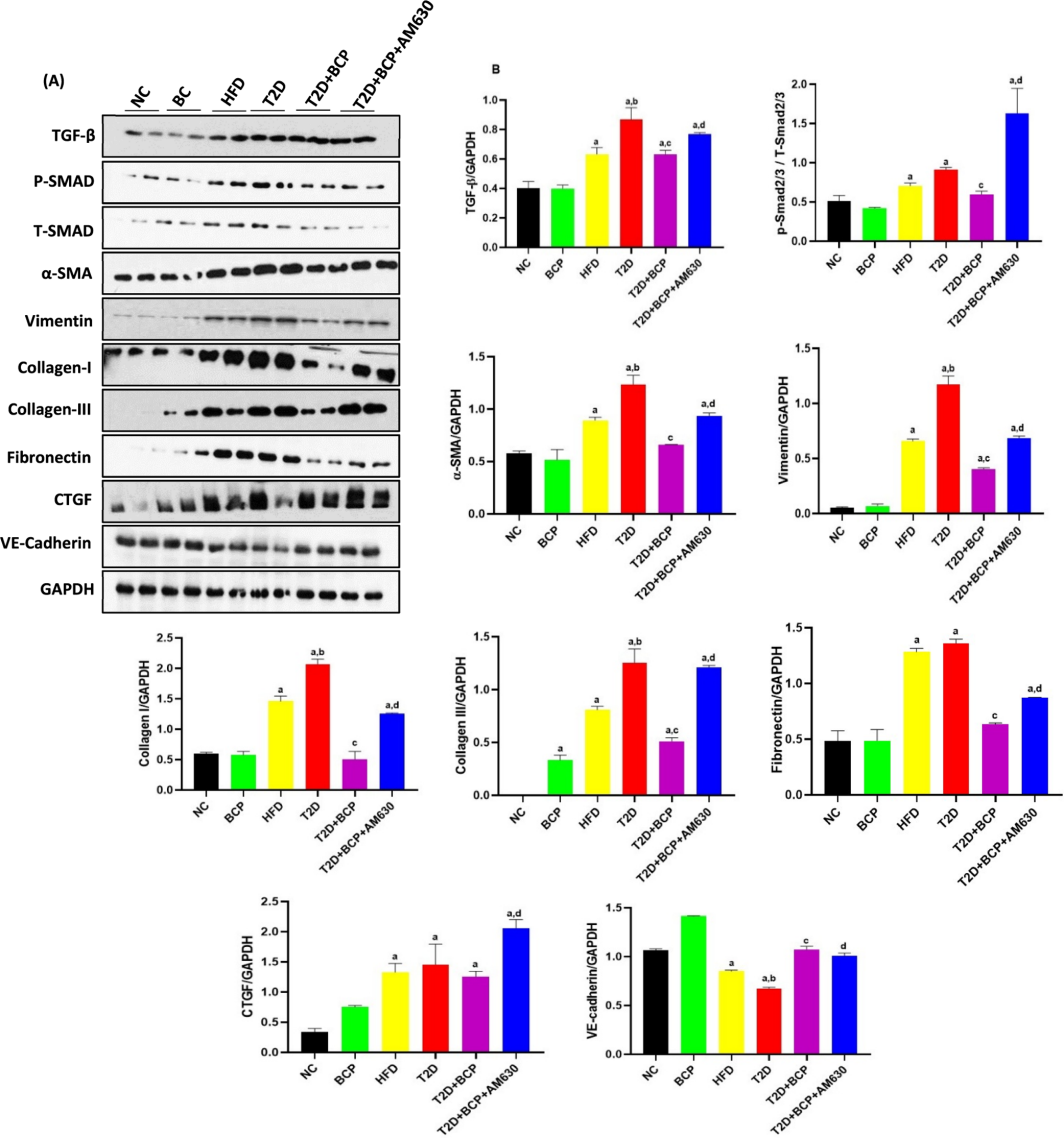
Effect of BCP treatment on hepatic fibrotic markers, EndMT, and TGF-β/Smad signaling in HFD/STZ-induced mice. (A) Western blotting analysis of TGF-β, p-Smad2/3, T-Smad2/3, α-SMA, vimentin, collagen I, collagen III, fibronectin, CTGF, and VE-cadherin. (B) Densitometric analysis of hepatic protein expressions of TGF-β, p-Smad2/3, T-Smad2/3, α-SMA, vimentin, collagen I, collagen III, fibronectin, CTGF, and VE-cadherin. Data are expressed as mean ± SEM (*N* = 3). Statistical analysis was performed using one-way ANOVA, followed by Tukey’s post hoc test. ^a^ *P* < 0.05 compared to NC, ^b^ *P* < 0.05 compared to HFD, ^c^ *P* < 0.05 compared to T2D, ^d^ *P* < 0.05 compared to T2D + BCP. TGF-β, transforming growth factor-β; Smad2/3, suppressor of mothers against decapentaplegic; α-SMA, α-smooth muscle actin; CTGF, connective tissue growth factor; VE-cadherin, vascular endothelial-cadherin.

CTGF, also named CCN2, is a regulatory molecule that plays a role in the production of the extracellular matrix.^37^ Increased levels of CTGF have been demonstrated in plasma obtained from patients with liver fibrosis and experimental models of hepatic fibrosis.^38,39^ In the current study, we observed an increased expression of CTGF in HFD mice, and the T2D group had more upregulated expression of CTGF, which was attenuated by BCP treatment. According to Figure 7A,B, these positive effects were nullified by the prior treatment with AM630. These outcomes collectively indicated that BCP had a considerable antifibrotic effect in T2D mice on a CB2-dependent mechanism.

### CB2R Agonist BCP Inhibits Hepatic EndMT via Attenuation of TGF-β/Smad Signaling in HFD/STZ-Induced Diabetic Mice

The expressions of endothelial marker, VE-cadherin, and mesenchymal markers, α-SMA and vimentin, were measured as indicators of endothelial-to-mesenchymal transition. The HFD animals showed reduced expression levels of endothelial markers (VE-cadherin) and enhanced expression levels of mesenchymal markers (α-SMA and vimentin), in comparison with the normal control mice. Further, the T2D mice showed a further reduction in endothelial marker expression and a greater increase in mesenchymal marker expression. BCP treatment in T2D mice showed prevention of the endothelial-to-mesenchymal transition (EndMT) phenotype. As depicted in Figure 7A,B, the role of BCP in the mitigation of EndMT transition was abrogated by the pretreatment with AM630. Thus, the activation of the CB2R by BCP can curb the hepatic EndMT transition in T2D mice.

Given the significant role of TGF-β/Smad signaling in regulating the EndMT transition and checking fibrosis, Western blotting was undertaken to determine their expression in hepatic tissues. The results showed that the expression of TGF-β and p-Smad2/3 to T-Smad2/3 ratio was substantially upregulated in HFD mice and further enhanced in T2D mice, which were then attenuated by BCP administration. However, AM630 administration before BCP reversed BCP’s protective effect (Figure 7A,B). Collectively, it was proposed that stimulation of the CB2R by BCP might relieve EndMT transition and hepatic fibrosis in T2D mice by inhibiting TGF-β/Smad signaling.

## Discussion

NAFLD has arisen as a remarkable health problem globally, specifically as a serious complication linked to T2D. The liver has a main regulatory function in maintaining glucose and lipid homeostasis. The metabolic dysregulation associated with T2D causes hepatic steatosis, which subsequently helps in developing many liver disorders.^40^ Currently, the majority of the therapeutics indicated for the management of diabetic liver damage, including NAFLD, are antihyperglycemic and hepatoprotective drugs, which reveal substantial therapeutic effects and obvious adverse effects.^41^ Appropriately, it is encouraging to uncover reliable compounds from medicinal plants to mitigate the diabetic liver injury.

In the current study, we utilized HFD/STZ-induced T2D mice to mimic the physiological situation of T2D patients linked with NAFLD. During the circumstances of insulin resistance triggered by T2D, insulin extremely enhances the production of lipids instead of decreasing glucose production, which ultimately results in hypertriglyceridemia and hyperglycemia.^42^ Extreme fat deposition can lead to liver damage and eventually cause NAFLD.^6^ The present study findings reveal that the serum levels of ALT, AST, and LDH were greatly increased in the diabetic animals. The observations of the current study are in line with previous studies.^10,43^ Intriguingly, the liver damage markers were clearly reversed after the BCP treatment. The beneficial effects of BCP were notably abolished by pretreatment with AM630, which indicates a CB2R-dependent mechanism of BCP.

Oxidative stress begins at an initial phase of diabetes and gets worse progressively, which is implicated in diabetic complications.^44,45^ The liver is considered a primary detoxifying organ and is responsible for the execution of oxidative processes, indicating that the liver is more vulnerable to alterations in the levels of oxidative damage in vivo. Oxidative stress happens as a result of the disparity between oxidant and antioxidant actions, which often results from increased release of free radicals and concomitant reduction in activities of antioxidants.^46^

Continuous high glucose, high fatty acid, and insulin production during the diabetic state can evoke ROS accumulation in cells, and excessive ROS interact with the macromolecules in the different organs, generating lipid peroxidation products, including MDA, that cause organ injuries.^44^ Some studies have demonstrated that the MDA was raised, and the antioxidant enzymes decreased in diabetic animals and NAFLD patients.^10,47^ It has been revealed that NADPH oxidases, NOX2 and NOX4, play a remarkable role in aggravating hyperglycemia in the liver in diabetic conditions.^48–50^ This proposes that the injury to the liver during diabetes can be ascribed to the ROS generated by NADPH oxidases. In agreement with previous reports, the liver of T2D mice showed a distinct rise in the MDA level, and expression of NOX2 and NOX4, coupled with reduced levels of GSH and activities of SOD and catalase.^51,52^ Herein, BCP treatment intensified the antioxidant condition and relieved oxidative stress in the T2D mice liver, proved by a decreased MDA level, NOX2 and NOX4 expression, and increased activities of GSH, SOD, and catalase. In agreement, BCP treatment showed antioxidant activities in different models of liver pathologies.^53,54^ The beneficial effects of BCP were attenuated by pretreatment with AM630, which distinctly reveals the CB2R-related mechanism in addition to the antioxidant property of BCP. The results presented demonstrate that BCP may mitigate diabetic liver damage by efficiently preventing oxidative stress in T2D mice via a CB2R-dependent mechanism.

A growing body of evidence recommends that Nrf2 signaling stimulation can protect hepatocytes from oxidative injuries and impede the development of NAFLD.^55^ HO-1, GPx1, and NQO1 are important antioxidant enzymes regulated by the Nrf2 pathway.^56^ In our study, we detected a significant fall in the expression of Nrf2 and downstream mediators like HO-1, GPx1, NQO1, SOD, and catalase concomitant with the upregulation of Keap1 expression in T2D mice. These results are consistent with earlier research.^52,57^ Interestingly, we found that Nrf2 was translocated into the nucleus, and the activity of Nrf2 was significantly elevated by BCP treatment. The target antioxidant enzyme expression of Nrf2, including HO-1, GPx1, NQO1, SOD, and catalase, was significantly enhanced, and Keap1 expression was downregulated by BCP treatment. BCP has been shown to exert hepatoprotective effects by favorable modulation of the Nrf2 signaling in diabetic complications.^35,58^ Nonetheless, the influence of BCP on antioxidant action was annulled when AM630 was injected before BCP treatment, revealing CB2R-mediated protective effects of BCP. The findings suggest that Nrf2 signaling is required for BCP to improve the HFD/STZ-induced oxidative injury of liver tissues.

Oxidative stress augmentation can prompt a series of cascade reactions, involving the activation of inflammatory signaling, which aggravates the liver damage.^10^ The T2D mice in this study showed an increase in levels of proinflammatory cytokines involving TNF-α, IL-6, IL-1β, and reduced anti-inflammatory cytokine IL-10 in liver tissues. Earlier studies described a positive connection between inflammatory cytokines and the development and progression of NAFLD.^59,60^ Treating T2D mice with BCP demonstrated a remarkable decrease in the liver proinflammatory cytokine and an increase in the anti-inflammatory cytokine. Remarkably, prior administration of AM630 resulted in a significant blockade of the protective effects of BCP, revealing a CB2R-associated mechanism underlying the anti-inflammatory effects of BCP.

The NLRP3 inflammasome is regarded as a crucial pro-inflammatory factor that is involved in the onset and progression of diverse pathologies.^61^ Usually, the activation of the NLRP3 inflammasome includes two-signal mechanisms. The first signal provokes the TLR4 signaling cascade, causing the upregulation of proinflammatory cytokines. Consequently, the second signal allows the NLRP3/ASC/pro-caspase-1 protein complex assembly that is essential for the activation of IL-1β and IL-18.^62^ Under diabetic conditions, enhanced levels of blood glucose, accumulation of free fatty acids, and overt generation of ROS amplify the stimulation of the NLRP3 inflammasome that leads to the onset of inflammation in numerous tissues.^61^ Previous studies have demonstrated that inhibition of activation of the NLRP3 inflammasome might offer benefits in diabetic liver injuries.^63,64^ The present study elucidates that NLRP3, ASC, and IL-18 expressions were remarkably upregulated in T2D mice, which was restrained considerably under the intervention of BCP. Previous studies showed that BCP mitigated inflammation by suppression of activation of the NLRP3 inflammasome.^35,65^ The prior administration of AM630 reversed these positive effects, indicating the apparent role of CB2R. Based on these results, we suggest that BCP alleviated inflammation in the liver of diabetic animals, mediating attenuation of NLRP3 inflammasome via CB2R activation.

Mechanistic studies demonstrate that BCP acts as a selective and specific agonist of the CB2R, a key regulator of immune and inflammatory responses. Activation of CB2R by BCP suppresses pro-inflammatory signaling pathways, including NF-κB and MAPK cascades, thereby mitigating the expression of proinflammatory cytokines, TNF-α, IL-1β, and IL-6. In addition to CB2R-dependent mechanisms, BCP exhibits antioxidant activity and modulates immune cell recruitment, collectively contributing to its broad anti-inflammatory profile. A recent mechanistic review further consolidates evidence supporting BCP’s therapeutic potential across multiple inflammatory and immune-mediated disease models, positioning BCP as a promising natural compound for targeting inflammation-driven pathologies.^66^

Hepatic fibrosis is a crucial phase in the progression of NAFLD, which is enhanced by augmented oxidative stress and inflammatory response.^67^ Upon liver damage initiated by oxidative stress or inflammation, hepatic stellate cells become stimulated and differentiate into myofibroblasts, which in turn results in the extensive accumulation of collagen and other components of the extracellular matrix, eventually culminating in hepatic fibrosis.^68^ The presence of collagen buildup in the liver tissues of T2D mice, along with increased expression of fibrotic markers, is suggestive of the role of fibrosis as an important contributor to the onset and progression of NAFLD. The present study observations are in line with previous reports, wherein the antifibrotic effect of BCP has been demonstrated in various organs.^69–72^ The above-mentioned effects were mitigated when diabetic mice were treated with BCP. Conversely, the prior treatment with AM630 abolished the antifibrotic activity of BCP, confirming the CB2R-dependent effects.

TGF-β is considered a potent inducer of EndMT and is a well-established cytokine that stimulates the profibrogenic signal and fibrosis in the liver.^73^ Our study is innovative, as it explores the effect of BCP-mediated CB2R activation on EndMT and endows a thorough insight into its antifibrotic mechanism. Our results demonstrated upregulated expression of fibroblast mediators and downregulated expression of endothelial mediators in T2D mice. Also, we noticed upregulation of TGFβ/Smad axis proteins in T2D mice. The administration of BCP salvaged this pathological transition; however, these positive effects were nullified when mice were pretreated with AM630, which is suggestive of the CB2R-dependent mechanism of BCP in ameliorating fibrosis.

A recent cheminformatics and machine-learning study identified that cannabis sativa-derived phytochemicals may modulate tissue microenvironments, oxidative balance, and fluid homeostasis through noncanonical targets, such as aquaporins, particularly aquaporin-4 (AQP4).^74^ While most work to date has focused on neurodegenerative contexts, these observations offer broader conceptual insight into how cannabinoid-related pathways may influence redox regulation, inflammation, autoimmunity, and tissue remodeling across organ systems, especially given AQP4’s role as an autoimmune target.^75^ Situating the present findings within this expanding mechanistic landscape would help align them with the wider scope of translational research for establishing cannabinoid based therapeutics.

Growing evidence indicates a complex crosstalk between oxidative stress, inflammation, and fibrosis pathways that involve Nrf2, NLRP3 inflammasome activation, and TGF-β signaling.^76,77^ The activation of Nrf2 decreases intracellular ROS, which acts as a crucial initiator for the priming and activation of the NLRP3 inflammasome, consequently restricting the subsequent stimulation of caspase-1 and the maturation of IL-1β and IL-18.^78^ Decreased oxidative stress and inflammasome activation might therefore lessen TGF-β/Smad-driven profibrotic signaling.^79^ β-Caryophyllene, via CB2 receptor activation, could function upstream by boosting Nrf2 signaling while inhibiting NF-κB-driven inflammation and ROS production, thus restraining NLRP3 activation and fibrotic signaling. Significantly, the reversal of BCP-mediated protective effects by AM630 in this study further underscores the regulation of oxidative stress, inflammasome activation, and profibrotic pathways through a specific receptor mechanism. Nonetheless, additional mechanistic investigations are necessary to elucidate the direct molecular interactions that connect CB2R signaling with the modulation of the Nrf2 and NLRP3–TGF-β axis.

## Limitations

The present study has several limitations that should be acknowledged. First, a T2D + AM630 alone group was not included in the experimental design. In this study, AM630 was primarily used as a pharmacological tool to confirm CB2R involvement in mediating the protective effects of BCP, rather than to investigate the independent metabolic effects of CB2R antagonism in T2D. Although inclusion of this group could provide additional insight into the direct contribution of CB2R signaling to disease progression independent of BCP treatment, the current design was optimized to specifically address CB2R-mediated mechanisms underlying BCP-induced protection. Future studies incorporating a T2D + AM630 alone group will help further delineate the independent role of CB2R signaling in metabolic and inflammatory alterations associated with T2D. Second, CB2R dependency was primarily assessed using the pharmacological antagonist AM630, which may exhibit off-target effects. Although our findings strongly support CB2R involvement, future studies employing CB2R knockout models or alternative selective antagonists are warranted to definitively confirm CB2R-mediated mechanisms. Third, while this study focused on oxidative stress, NLRP3 inflammasome activation, and TGF-β/Smad signaling, other key pathways implicated in NAFLD progression, including dysregulated lipid metabolism and endoplasmic reticulum stress, were not evaluated. Future investigations exploring these pathways will provide a more comprehensive understanding of NAFLD pathogenesis and BCP-mediated hepatoprotection. Finally, the study was conducted exclusively in male animals, limiting the evaluation of sex-specific responses, and clinically translatable biomarkers were not validated. Addressing these aspects in future studies will further enhance the mechanistic depth and translational relevance of the present findings.

## Data Availability

The data used to support the findings of this study are already incorporated in the results section.
